# The actin multigene family of *Paramecium tetraurelia*

**DOI:** 10.1186/1471-2164-8-82

**Published:** 2007-03-28

**Authors:** Ivonne M Sehring, Jörg Mansfeld, Christoph Reiner, Erika Wagner, Helmut Plattner, Roland Kissmehl

**Affiliations:** 1 University of Konstanz, Department of Biology, P.O. Box 5560, 78457 Konstanz, Germany; 2 present address: Institut f. Biochemie, Schafmattstr. 18, ETH-Hönggerberg, HPM F 8, 8093 Zürich, Switzerland

## Abstract

**Background:**

A *Paramecium tetraurelia *pilot genome project, the subsequent sequencing of a Megabase chromosome as well as the *Paramecium *genome project aimed at gaining insight into the genome of *Paramecium*. These cells display a most elaborate membrane trafficking system, with distinct, predictable pathways in which actin could participate. Previously we had localized actin in *Paramecium*; however, none of the efforts so far could proof the occurrence of actin in the cleavage furrow of a dividing cell, despite the fact that actin is unequivocally involved in cell division. This gave a first hint that *Paramecium *may possess actin isoforms with unusual characteristics. The genome project gave us the chance to search the whole *Paramecium *genome, and, thus, to identify and characterize probably all actin isoforms in *Paramecium*.

**Results:**

The ciliated protozoan, *P. tetraurelia*, contains an actin multigene family with at least 30 members encoding actin, actin-related and actin-like proteins. They group into twelve subfamilies; a large subfamily with 10 genes, seven pairs and one trio with > 82% amino acid identity, as well as three single genes. The different subfamilies are very distinct from each other. In comparison to actins in other organisms, *P. tetraurelia *actins are highly divergent, with identities topping 80% and falling to 30%. We analyzed their structure on nucleotide level regarding the number and position of introns. On amino acid level, we scanned the sequences for the presence of actin consensus regions, for amino acids of the intermonomer interface in filaments, for residues contributing to ATP binding, and for known binding sites for myosin and actin-specific drugs. Several of those characteristics are lacking in several subfamilies. The divergence of *P. tetraurelia *actins and actin-related proteins between different *P. tetraurelia *subfamilies as well as with sequences of other organisms is well represented in a phylogenetic tree, where *P. tetraurelia *sequences only partially cluster.

**Conclusion:**

Analysis of different features on nucleotide and amino acid level revealed striking differences in isoforms of actin and actin-related proteins in *P. tetraurelia*, both within the organism and in comparison to other organisms. This diversification suggests unprecedented specification in localization and function within a unicellular eukaryote.

## Background

Actin is described as one of the most abundant and highly conserved proteins in eukaryotic cells. It may be present as monomeric G-actin or as filamentous F-actin and is involved in many vital cellular functions such as organelle transport [1], cell motility (reviewed in [2]), cytokinesis [3], cytoplasmic streaming in plants [4], in regulating trafficking of membrane proteins like the vacuolar H^+^-ATPase [5], in exocytosis [6] and in different steps during endocytosis [7], phagosome/lysosome fusion [8,9] and post-Golgi transport [10]. Nuclear actin is involved in transcription (reviewed in [11]). This functional diversification may be accounted for by the molecular diversification of actin in the ciliated protozoan, *Paramecium tetraurelia*, where immuno-localization has revealed numerous sites of actin enrichment [12].

The number of actin genes in a species can widely vary [13]. Multicellular organisms have several isoforms of cytoplasmic actin, which are coexpressed in most cell types and share very similar sequences with each other [14]. In addition, a range of actin-like (ALP) and actin-related proteins (ARP) exist, which are conserved across all eukaryotes [15-17]. ARP functions range from specialized effects on conventional G- and F-actin structures to structural roles that are apparently independent of actin; for example, ARP1 is a main component of the dynactin complex [17]. Actins, ALPs and ARPs define a large family of homologous proteins, the actin superfamily, which share the same structural architecture, known as the "actin fold", and an overall sequence similarity to actin [18]. The actin fold is functionally characterized as an ATPase domain with ATP-binding capacity in the presence of Mg^2+ ^or Ca^2+^. Monomeric actin binds ATP, which is hydrolyzed to ADP after incorporation of the actin monomer into a filament. This hydrolysis is important for the dynamic turnover of actin filaments. Also for several ARPs, hydrolysis of bound ATP is necessary for their function [19].

A brief look into close relatives of *Paramecium *shows wide variability. Although in most eukaryotes actin is encoded by a multigene family, there are also organisms where only one single actin gene is described so far, e.g. the parasitic apicomplexans, *Toxoplasma gondii *and *Cryptosporidium parvum *[20,21], i.e. close relatives of ciliates. Several studies on ciliate actin showed that it is different from that of other eukaryotes and therefore described as "unconventional" [reviewed in [22]].

In *P. tetraurelia*, a first characterization of actin on the molecular level was achieved when Diáz-Ramos et al. (1998) cloned an actin gene fragment of 1,138 bp, i.e., more than 96% of the coding sequence of a standard actin gene [23]. It was called α-actin as it had the highest sequence identity to that form in other organisms. Sequence data provided by a pilot sequencing project [24,25], the sequencing of a macronucleic one-megabase chromosome [26] and a current genome project at Genoscope [27] allowed us to search the *P. tetraurelia *genome for further actin genes. We found 30 genes for actin, ARPs and ALP, which could be grouped in 12 widely diverging subfamilies, with varying intron numbers and positions, ATP-, myosin II- and drug-binding sites.

## Results

### Definition of actin genes and subfamilies

#### Actin1 subfamily

In order to complete the missing ends of the first published actin gene of *P. tetraurelia *[23], we took advantage of an indexed genomic library [27] by using a ~ 1 kb probe designed from the sequence of this actin gene [Genbank:X94954]. By performing a two step hybridization, several clones were retrieved and sequenced. One of them, clone 87M3, corresponds to the incomplete actin sequence previously published [23]. It contains the entire actin sequence including the missing 5'- and 3'-ends, but also 18 nucleotide substitutions (when compared to the sequence published by Diáz-Ramos *et al*. [23]) predominantly at the ends of the sequence. This actin gene, actin 1-1 [Genbank:AJ537442], consisting of 1128 bp, encodes a protein of 375 amino acids (aa), with a calculated molecular weight of 41,700 (Table 1). We also have cloned this gene from the cDNA synthesized from the total RNA of vegetative cells. The coding sequence is interrupted by two short introns that display the characteristics of *P. tetraurelia *introns, i.e., they are bordered by 5'-GT and AG-3' and 21–31 nucleotides long.

By sequencing the other 6 clones, we found two other isoforms, actin1-2 [Genbank:AJ537443] and actin 1-3 [Genbank:AJ537444], which differ from actin1-1 by 4 to 8% on the nucleotide level (Table 1). However, on the amino acid level all three actins are identical and they all contain the two introns at the same position. The presence of all three genes in a cDNA library indicates that all three isoforms are expressed. For other actin subfamily 1 members, see below.

#### Actin2 subfamily

Among the 722 protein encoding genes identified in the course of the pilot sequencing project of the *P. tetraurelia *macronuclear genome we also found a partial sequence with homology to another mammalian actin. Sequencing of the corresponding clone, M07D05u, resulted in the identification of the 5'- end of this gene. To obtain the missing 3'- end, we took advantage of the indexed genomic library [28], which we analyzed in two sequential hybridization steps by using a specific probe designed from sequence M07D05u. Among nine clones, 5 contained the complete sequence information of this gene and 4 that of a closely related actin isoform. The two genes were called actin2-1 [Genbank:AJ537446] and actin2-2 [Genbank:AJ537447]. Sequencing from a *Paramecium *cDNA-library revealed the cDNA information of the corresponding genes. The ORFs, which are interrupted by 5 short introns, encode proteins of 376 amino acids with a calculated molecular mass of 42.4 kDa (Table 1).

#### Other actin subfamilies

During early steps of the *P. tetraurelia *whole genome shotgun sequencing undertaken by Genoscope, and by manual assembly of single reads extracted from the draft assembly we used, we were able to identify further actin coding genes, resulting in a total number of 30 genes encoding actins, ARPs and ALP. They can be classified into 12 subfamilies, according to their sequence identity, their length, and the number of introns as well as their position.

Interestingly, the megabase chromosome sequencing project [25] revealed a cluster of three actin genes (scaffold1; accession number CR548612; actin1-6, actin3-2 and actin8-1). On the related sister scaffold (scaffold 8) only two actins were present (actin1-7, accession number CR855973, and actin3-1, accession number AJ537448). The incidence of two closely related isoforms is probably due to recent whole genome duplication in *P. tetraurelia *(J. Cohen and L. Sperling, personal communication; [29]).

#### General features of *Paramecium *actin sequences

**Figure 1:**
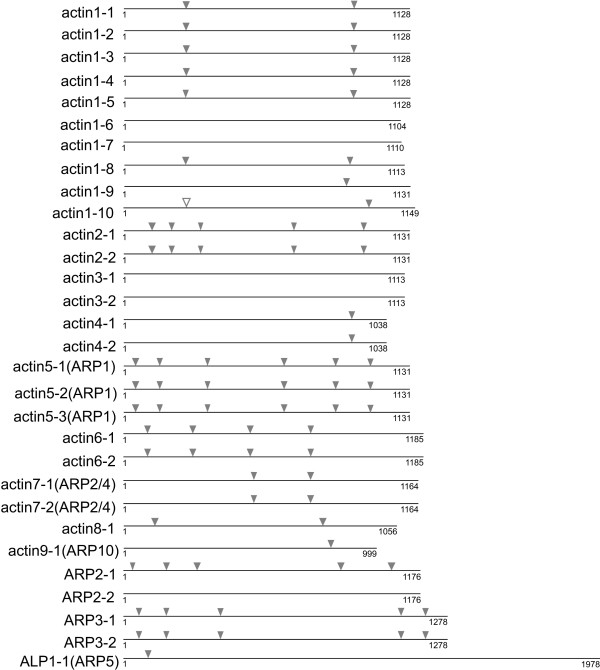
**Position of introns in the nucleotide sequence of actins and actin-related proteins in *P. tetraurelia***. The position of the introns (gray arrowhead) are as follows (bp): subfamily 1, intron I (241–270), intron II (907–933); subfamily 2, intron I (110–140), intron II (218–243), intron III (332–354), intron IV (731–754), intron V (976–1001); subfamily 4, intron I (900–925); subfamily 5, intron I (45–74), intron II (139–163), intron III (359–381), intron IV (690–716), intron V (922–948), intron VI (1108–1132); subfamily 6, intron I (93–120), intron II (273–303), intron III (530–528), intron IV (799–825); subfamily 7, intron I (514–539), intron II (738–766); subfamily 8, intron I (121–141), intron II (780–804); subfamily 9, intron I (818–845); subfamily ARP2, intron I (35–57), intron II (169–189), intron III (321–350), intron IV (920–949), intron V (1129–1161); subfamily ARP3, intron I (58–82), intron II (168–191), intron III (410–435), intron IV (1170–1196), intron V (1280–1305); subfamily ALP1, intron I (84–109). White arrowhead marks non-spliced intron (see text).

Based on an alignment of all 30 actin-related genes found in *P. tetraurelia *(ClustalW alignment, see additional file 1), they can be divided into twelve subfamilies (Table 1): seven pairs, one trio, three single genes, and a large subfamily with 10 members, one of which seems to be a pseudogene (see below). Within a subfamily, proteins are of equal size. Exceptions are the members of the largest subfamily 1 which vary between 370 and 376 amino acids. While several *P. tetraurelia *actin genes encode proteins with the common actin length of 375 aa, there are also some smaller (down to 332 aa) and some larger ones (up to 394 aa). Both ARP2 and ARP3 isoforms, with 391 aa and 426 aa, respectively, are in the range of the usual length for those proteins. Additionally one gene encodes a large actin-like protein with 658 aa (Table 1). Amplification with gene-specific primers (see additional file 2) from *P. tetraurelia *cDNA indicates the expression of selected isoforms and reveals cDNA information about these genes. In detail, comparison of these sequences with the genomic version allows us to determine number, size and position of the introns. The number and positions of introns varies between the different subfamilies, while they are identical within a subfamily (Fig. 1). An exception is again subfamily 1, where six members possess two introns, while two members contain only one, and two members none. In subfamily 3, the two members do not contain any introns. Subfamily 5, where the open reading frame (ORF) is interrupted by six introns, has the highest number of introns (Fig. 1, Table 1). For actin1-10, re-sequencing from cDNA confirms that within this part of the SuperContig sequence a conserved 26 bp-intron contains an unusual 3' border ('ttg' instead of a 'tag') and therefore cannot be spliced. As a consequence a frame shift occurs, causing a termination signal and a truncated protein (103 aa).

Members within a subfamily share more than 80% sequence identity at the nucleotide level (Table 1; alignment with Clustal W). Exceptions are the members of subfamily 1 whose amino acid composition varies by up to 34%. The different actin subfamilies are highly divergent from each other. Comparing the amino acid sequence of all of them to actin1-1, the isoforms of the actin2 subfamily have the highest identity with less than 60%. Members of other subfamilies have less than 50% identity, with the most diverse isoform, actin9-1, sharing only 17.6% identity. Actin1-1 was chosen as reference sequence as it is, together with the identical (on amino acid level) isoforms actin1-2 and actin1-3, the most conserved actin in comparison to actins from other organisms (Table 2). Nevertheless, even these isoforms share less than 80% identity with actins of selected model organisms. This wide diversification of *P. tetraurelia *actins is manifested in the differences found in several functional characteristics, as shown below.

#### Actin-related proteins (ARPs)

For some *P. tetraurelia *actin isoforms, NCBI Blast searches leads to hits for both actins and ARPs of other organisms. Therefore, we additionally used ARPAnno, an actin-related protein annotation server designed by Muller *et al*. [18], for classification of our sequences. In general, the results from NCBI Blast search and ARPAnno concur, only in two cases different classifications were obtained (see below). According to ARPAnno results, actin5 might be classified as ARP1 (score: 47.5; score for actin: 44.0; score ranging from 0 to 100). For ARP1, three isoforms (actin5-1, actin5-2 and actin5-3) exist, while both ARP2 and ARP3 are represented by two isoforms. The nuclear ARPs, ARP4 and ARP6, which are copresent in certain chromatin remodeling complexes, are omnipresent in eukaryotic organisms, except for the parasitic protozoan, *Encephalitozoon cuniculi *[18]. Similarly, in the *P. tetraurelia *genome, we only found putative orthologs for ARP4 (actin7) but none for ARP6. The designation as ARP4 is due to blast search hits for ARP4 from the NCBI database. In contrast, alignment with ARPAnno gave the best score for ARP2 (29.1), but the score for ARP4 is just slightly lower (28.8), just as the score for actin (27.0). For the single ALP found in the *Paramecium *database, NCBI blast search showed also hits with nuclear ARP5, which is supported by a best ARPAnno score for ARP5 (28.7; score for actin: 26.3). For nuclear ARP8, we could not find any ortholog. The functionally obligate heterodimeric partners ARP7 and ARP9, the two ARPs restricted to fungi so far [18], are not present. Actin9, the most divergent isoform in *P. tetraurelia*, is difficult to classify due to different results obtained with BlastP searches and ARPAnno alignments (actin/ARP10 and ARP2 (score: 24.8)/orphans [sequences lacking common defining characteristics, score: 24.8; score for actin: 23.2], respectively).

#### Phylogenetic distribution

**Figure 2:**
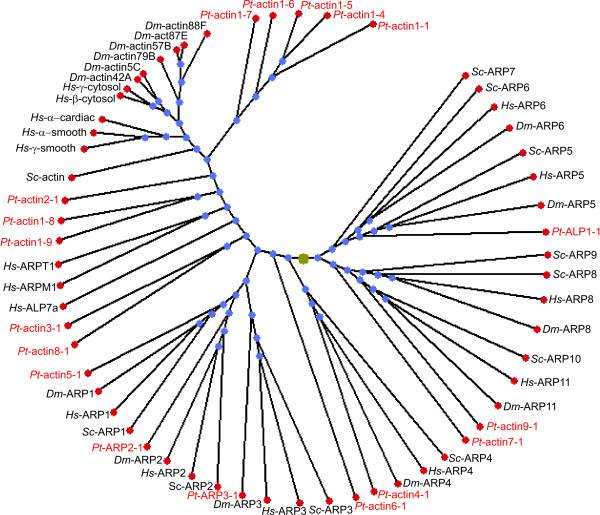
**Phylogenetic tree of actins and ARPs**. This encompasses *Drosophila melanogaster *actin42A [GenBank:CG12051], actin57B [GenBank:Q53501], actin5C [GenBank:Q6YN46], actin79B [GenBank:P02574], actin87E [GenBank:Q8MZ23], actin88F [GenBank:P83967], ARP1 [GenBank:P45889], ARP2 [GenBank:P45888], ARP3 [GenBank:P32392], ARP4 [GenBank:Q9V814], ARP6 [GenBank:P83967], ARP11 [GenBank:Q9VWE8], ARP8 [GenBank:Q9VX09], ARP11 [GenBank:Q9VWE8]; *Homo sapiens *cardiac α-actin [GenBank:P04270], smooth α-actin [GenBank:P03996], cytosol. β-actin [GenBank:P60709], cytosol. γ-actin [GenBank:AAH15779], smooth γ-actin [GenBank:P12718], ARP1 [GenBank:P61163], ARP2 [GenBank:P61160], ARP3 [GenBank:AAH44590], ARP4 [GenBank:O96019], ARP5 [GenBank:Q8IUY5], ARP6 [GenBank:Q9GZN1], ARP8 [GenBank:Q9H981], ARP11 [GenBank:Q9NZ32], ARPM1 [GenBank:Q9BYD9], ARPT1 [GenBank:Q96L10], ALP7β [GenBank:Q9Y614]; *Paramecium tetraurelia *act1-1 [GenBank:CAD60960], act1-4 [GenBank:CAD60963], act1-5 [GenBank:CAH69678], act1-6 [GenBank:CAH03399], act1-7 [GenBank:CAH69677], act1-8 [GenBank:CAH69676], act1-9 [GenBank:CAH69752], act2-1 [GenBank:CAD60964], act3-1 [GenBank:CAD60966], act4-1 [GenBank:CAH69675], act5-1(arp1-1) [GenBank:CAH69674], act6-1 [GenBank:CAH69671], act7-1 (arp4-1) GenBank:CAH74221], act8-1 [GenBank:CAH03397], act9-1 (arp10) [GenBank:CAH69669], alp1-1 (arp5) [GenBank:CAH69680], arp2-1 [GenBank:CAH69679], arp3-1 [GenBank:CAH74222]; *Saccharomyces cerevisiae *actin [GenBank:P60010], ARP1 [GenBank:P38696], ARP2 [GenBank:P32381], ARP3 [GenBank:P47117], ARP4 [GenBank:P80428], ARP5 [GenBank:P53946], ARP6 [GenBank:Q12509], ARP7 [GenBank:Q12406], ARP8 [GenBank:Q12386], ARP9 [GenBank:Q05123], ARP10 [GenBank:Q04549]

The growing number of sequence data from different organisms available allows us to investigate the phylogenetic distribution of *P. tetraurelia *actins and actin-related proteins (Fig. 2). A phylogenetic tree with all actins and ARPs of a set of sequenced genomes (*P. tetraurelia*, *H. sapiens*, *D. melanogaster *and *S. cerevisiae*) was created (Fig. 2) using PhyloDraw [30], (matrix: neighbor joining). For a clear arrangement, we have selected one member of each *P. tetraurelia *subfamily; an exception is subfamily one, where only the isoforms actin1-2 and 1-3 were excluded, as they are identical to actin1-1 at amino acid level. The first members of subfamily 1 cluster in one branch, while actin1-8 and 1-9 group together in another branch. Subfamilies 3 and 8 build a similar small branch, while subfamily 2, 6 and 7 are single-standing. None of them cluster together with any sequence from other organisms. However, *P. tetraurelia *ARP3 clusters with ARP3 from other organisms, as does ARP2. Likewise, *P. tetraurelia *actin5, actin9 and ALP1 sequences, which all could be classified as ARPs (ARP1, ARP2/10 and ARP5, respectively), cluster in branches with the respective ARP sequences from the other organisms. The high diversity of actin genes even within protozoa is indicated in a phylogenetic tree composed of actin and ARP sequences from 13 different protozoa (see additional file 3). The overall distribution of *P. tetraurelia *sequences in both trees is similar. However, with an expanded tree, using 71 sequences from 26 organisms, the clustering of possible *P. tetraurelia *ARPs with ARPs from other organisms is clearly reduced (see additional file 4). This observation was verified by a phylogenetic tree with 40 additional sequences, which resulted in the same isolated branches for *P. tetraurelia *sequences (data not shown).

#### Actin consensus pattern

**Figure 3:**
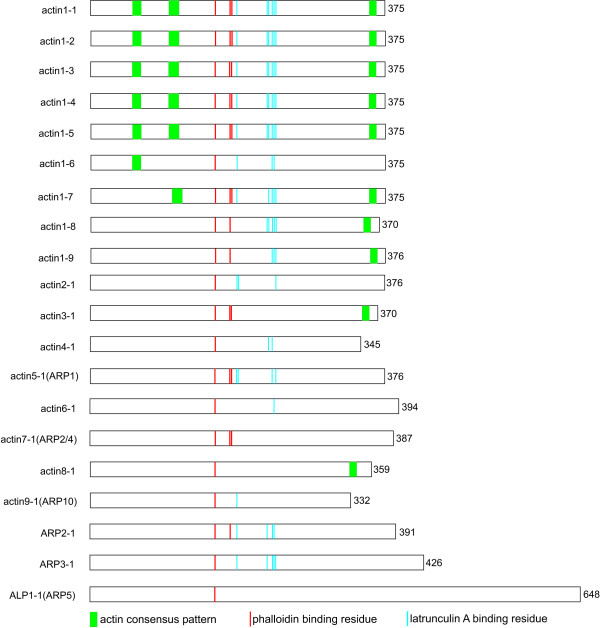
**Characteristic features of *P. tetraurelia *actins, ARPs and ALP**. Characteristic features include consensus and drug binding motifs. These are identical within subfamilies wherefore only one isoform of each is shown as an example. The actin signatures (green) are based on the three actin consensus patterns according to PROSITE: ACTINS_1, [FY]-[LIV]-G-[DE]-E-A-Q-x-[RKQ](2)-G; ACTINS_2, W-[IV]-[STA]-[RK]-x-[DE]-Y-[DNE]-[DE] and ACTINS_3, [LM]-[LIVM]-T-E-[GAPQ]-x-[LIVMFYWHQ]-N-[STAQ]-x(29-N-[KR]. Not all isoforms contain the typical consensus pattern found in most of the actin genes described in other species. Also the three residues mediating the binding of phalloidin (G158, R177 and D179) are not present in every subfamily (red). The same is true for the residues (blue) relevant for the binding of latrunculin A (D157, R183, D184, R210, D211, K213, E214 and K215).

PROSITE [31] has developed three signature patterns which detect most of the sequences known to belong to actin. Two of them (ACTINS_1, [FY]- [LIV]-G- [DE]-E-A-Q-x- [RKQ](2)-G, position 54 to 64, and ACTINS_2, W- [IV]- [STA]- [RK]-x- [DE]-Y- [DNE]- [DE], position 357 to 365) are specific for actins, while one signature (ACTINS_3, [LM]- [LIVM]-T-E- [GAPQ]-x- [LIVMFYWHQ]-N- [STAQ]-x(29-N- [KR], position 106 to 118) selects actins, ARPs and ALPs. Most actin subfamilies, namely 2, 4, 5, 6, 7, 9, the ARPs and ALP, do not possess any actin consensus pattern (Fig. 3). Actin signatures are mainly present in the actin1 subfamily, where every isoform has at least one consensus pattern, but only the isoforms actin1-1 to actin1-5 have all three of them. The ACTINS_1 and ACTINS_3 signatures are restricted to the actin1 subfamily. The ACTINS_2 signature is the most common, with an expression in nearly all isoforms of the actin1 subfamily (except for actin1-6), in the actin3 subfamily and in actin8-1.

#### Amino acids influencing polymerization

Several specific amino acids were proven to be involved in subunit interaction across the actin filament. An intermolecular coupling of the DNase I binding loop (residues 38–52) and the C terminus [32], and of the hydrophobic plug (residues 262–274) and the C terminus [33] has been suggested. Examples of intermonomer cross-linking of F-actin are the residue pairs H40/E167, Q41/C374, S265/C374, and Q41/C265 [34,35]. We aligned the *P. tetraurelia *actin sequences with a number of actins from other organisms (including those in Table 2, and in addition, another *H. sapiens *actin sequence [Genbank:CAG38753] and a *P. falciparum *sequence [Genbank:PFL2215w]) and checked for the presence of the above-mentioned residues. Most isoforms do not possess a pair of interacting amino acids (Table 3). Only in subfamily 1 we could find such pairs. This is mainly due to the lack of the residues in the DNase I binding loop, H40 and Q41, respectively, in all other subfamilies. In most subfamilies, the amino acid conservation in the DNase I binding loop is generally weak (data not shown). The same holds true for the hydrophobic plug. The amino acid S265, which interacts intermolecularly with two residues, is only present in actin1-8.

#### ATP binding

**Figure 4:**
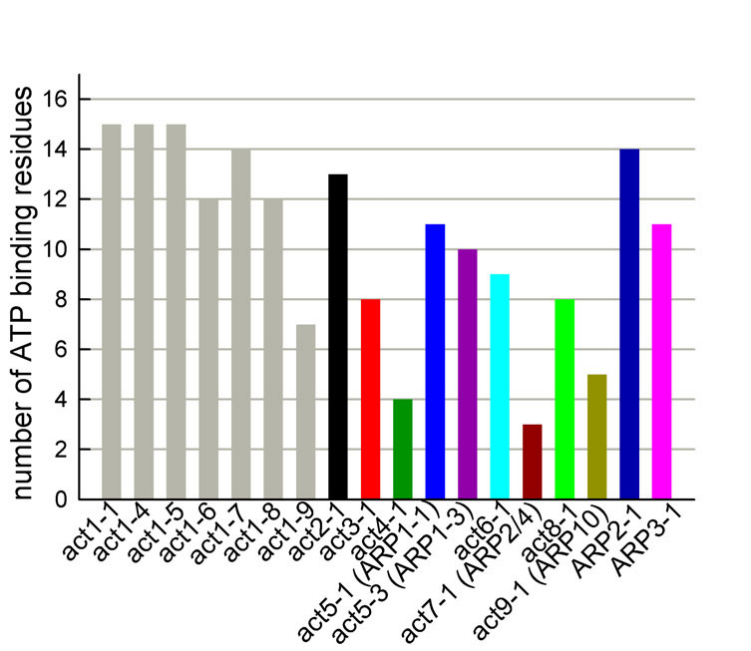
**Conservation pattern of residues involved in ATP binding.**Conservation pattern of 17 residues (D13, S16, G17, L18, K20, Q139, D156, D159, G160, V161, K215, G304, T305, M307, Y308, and K338) known to participate in ATP binding to actin. For subfamilies in which the conservation pattern is identical for all members, only one is shown.

Both actin and ARPs bind ATP. The hydrolysis of ATP to ADP is proposed to induce a conformational change required for their biological function [19,36]. The conservation of 17 key reference residues involved in nucleotide binding was analyzed (Fig. 4). The isoforms can be clustered in three groups. The first group is composed of actin1-1 to actin1-8, the subfamily actin2, actin5-1, actin5-2, and the ARP subfamilies 2 and 3, all possessing > 60% conserved residues. The second group, composed of actin1-9, the subfamilies actin3, actin5-3, actin6, and actin8, has 40–60% conserved residues. The third group displays a very low level of conserved residues with less than 30% (subfamilies actin4, actin7 and actin9).

#### Binding residues for myosin II

**Figure 5:**
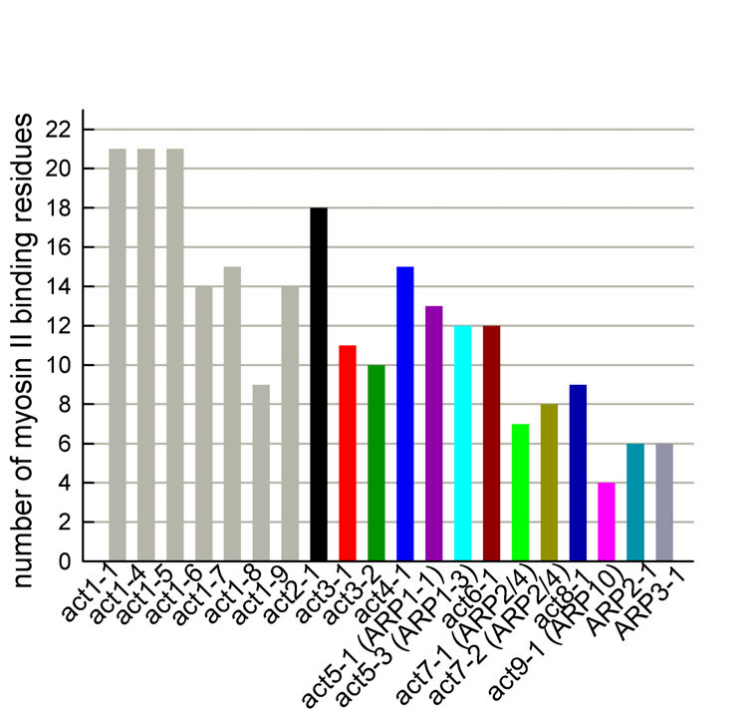
**Conservation pattern of myosin II- binding residues.**Conservation pattern of 23 myosin II-binding residues (2, 24, 25, 40, 42, 144–148, and 341–353). For subfamilies in which the conservation pattern is identical for all members, only one is shown.

Actin binds a substantial number of proteins collectively called actin-binding proteins (ABP). One of the best studied is myosin II. We looked for the expression of myosin II binding residues [37] in *P. tetraurelia *(Fig. 5). The binding site is well conserved in subfamilies 1 (except act1-8), 2 and 4. In several members of subfamily one, all residues are present. Most of the other subfamilies show at least 40% identity, only in some actin/ARP subfamilies the conservation is weak.

#### Drug binding residues

Several drugs can interact with actin where they interfere with polymerization and depolymerization. Phalloidin binds to actin filaments and stabilizes them against depolymerization [38]. Using fluorescently labeled phalloidin is a common approach to visualize actin filaments. Since mutagenesis studies have identified drug binding sites, we had a closer look at phalloidin binding sites. As such, G158, R177 and D179 have been suggested [39,40]. Using the multiple sequence alignment of actins, we found that only six actin isoforms possess all three of these amino acids (Table 4, Fig. 3). While G158 is highly conserved and exists in all isoforms, the other two residues are lacking in many subfamilies.

Latrunculin is another actin binding drug for which mutagenesis studies revealed the binding residues. This drug sequesters actin monomers by making 1:1 complexes [41]. In yeast mutagenesis studies, three mutated alleles (act1-112: K213, E214, K215; act1-113: R210, D211; act1-117: R183, D184) lead to a complete resistance to latrunculin A [42]. Analysis of *P. tetraurelia *actin sequences showed that the residues for latrunculin A binding are mainly present in the actin1 subfamily, but no isoform possesses all of them (Table 4, Fig. 3). Only sporadic binding residues can be found in the other subfamilies, and none in actin subfamilies 3, 7 and 8. Amino acid D157, which has also been implicated in the binding of latrunculin A [40], is not present in any of the *P. tetraurelia *actins (Table 4).

## Discussion

Extensive *Paramecium *database searches and analyses allowed us to identify 30 genes in *Paramecium *as actins, actin-related proteins or actin-like proteins, according to NCBI Blast hits. They cluster in 12 subfamilies with varying intron positions and numbers, which range from zero to six (Table 1). Genes encoding actin have been cloned also in several other protists [43], including apicomplexans [44], which, together with ciliates such as *Paramecium *and *Tetrahymena*, belong to the phylum Alveolata. In *Cryptosporidium*, the actin gene is intronless, and in *Toxoplasma *it has one intron. In *Plasmodium falciparum*, another Apicomplexan, there are two genes encoding actin [44]. One gene (actin I) is intronless and is expressed throughout the parasite life cycle, while the actin II gene has one intron and is transcribed only in the sexual stages.

### Identification of ARPs and ALPs

The amino acid sequence variation between the different *P. tetraurelia *actin subfamilies is very high. When compared to actin1-1, which is most similar to conventional actins, most of them show less than 50% identity. In the literature, ARPs share between 17 and 60% amino acid identity with conventional actins [16]. Due to contradictory hits with blast searches in the database, some *P. tetraurelia *subfamilies could be assigned to both, actins or ARPs. Most "double-hits" are confirmed by alignments with ARPAnno. However, the scores obtained with ARPAnno for their respective ARP classification were below the value which was determined as highly reliable by the designers (score > 55), and only slightly higher than the scores for actin. Therefore, their assignment to an established ARP family is critical and we rely on the NCBI Blast search results for their classification. Hence, the respective proteins are annotated as actins in the *Paramecium *database, with the indication ARP in brackets.

From the eleven ARP subfamilies described in the NCBI database, six have presumably orthologs in *P. tetraurelia*. The lack of several subfamilies is not unexpected as some of them are restricted to, or absent from different phyla [18]. ARP1 (also called centractin), a major component of the dynactin complex, is present in a wide range of organisms, from yeast to humans, but absent from *Arabidopsis*, rice and possibly from other plants [18]. Yeast has a single ARP1 gene, whereas higher eukaryotes have at least two or perhaps three isoforms [16]. In *P. tetraurelia*, the three members of the actin5 subfamily might be orthologs of ARP1. They match the general length of ARP1 in vertebrates (376 aa), but they are less identical to conventional actins than in general (~ 40% in comparison to 55–60% [45]).

Besides ARP1, a second actin-related protein is found in the dynactin complex – ARP10 (yeast) or ARP11 (fly and vertebrates). According to NCBI database blast, actin9 could be an ortholog of ARP10. However, ARPAnno alignment resulted in an equally low score for ARP2 and orphans, which makes an accurate classification difficult. ARP2 and ARP3 form the ARP2/3 complex, which is a major nucleator of actin polymerization [46]. Both ARPs are present in *P. tetraurelia*, while they are both absent from apicomplexans [47]. Additionally we found sequences which are presumably orthologs of the nuclear ARP4 (actin7) and ARP5 (ALP1). Admittedly, the classification of actin7 as ARP4 according to NCBI blast search might be critical, as ARPAnno gave a slightly better score for ARP2. Both, ARP4 and ARP5, are thought to be copresent in chromatin remodeling complexes with some other ARPs. Surprisingly we could not find orthologs of the respective partners (ARP6 and ARP8) in the database. ARP4 and ARP6 are the most conserved ARPs regarding the distribution among the eukaryotic phyla, while both ARP5 and ARP8 are absent from several organisms [18]. It would be interesting to determine if the lack of these ARPs correlates with a lack of the corresponding complementary subunit, or if their functions are fulfilled by other proteins. However, despite extensive search in the fully covered *Paramecium *database, we cannot definitely exclude that potential genes may have escaped our data mining approach. This could be due to high evolution rates and/or disruption by many or unconventional introns.

### Actin isoforms

The high number of actin and ARP sequences in *P. tetraurelia *seems to be quite unusual for a unicellular organism. While for several multicellular organisms, an exorbitant number of actin genes was reported (for example, the flowering plant *Petunia *was shown to have over 100 actin genes [48]), the situation in protists seems to be rather different. Several genome projects (*Leishmania major, Plasmodium berghei, Plasmodium falciparum, Trypanosoma brucei *and *Tetrahymena thermophila*) revealed only about 10 sequences related to the actin superfamily. However, in the slime mold, *Dictyostelium discoideum*, also over 30 genes for actin and ARPs have been found [49].

Several possible reasons were discussed why organisms have multiple actin isoforms [50]. One argument is that, as actin plays a role in many cellular processes, organisms need a large quantity of actin, and the best way to provide enough actin may be to have multiple genes. In fact, in *P. tetraurelia*, we found three isoforms which are identical on amino acid level (actin1-1, 1-2 and 1-3), and they are all expressed. While their existence could be explained with genome duplications in *P. tetraurelia *[29], the expression of all three of them might be a tool for gene amplification. A related explanation is derived from the fact that, in *Drosophila*, two cytoplasmic actin genes show a differential temporal and spatial expression [51]. Finally, amino acid difference may be important, allowing the different isoforms to exert different roles in the same cell. The high degree of divergence observed in *P. tetraurelia *actins might be a hint to the last hypothesis. This may be exceptional for an exceptionally complex cell. In contrast, in the model plant *Arabidopsis*, there are eight actin genes, which can be divided into vegetative and reproductive classes, and are expressed in a tissue-specific manner [52]. The spectrum of actin proteins may reflect the mixture of actin functions and/or patterns of regulation in different plant organs [48]. But also within a single cell, specialized function could be observed. For instance, within muscle cells, muscle actins may be preferentially utilized for the formation of myofibrils, whereas cytoplasmic actin isoforms may be used exclusively for some cytoskeletal functions not related to contraction [52]. Several studies in other systems also suggest that different isoforms do have specialized functions [14]. This may also be assumed for the numerous isoforms we find in *P. tetraurelia*, considering the multiple localization sites [12,53].

Another interesting point is the possible redundancy of various isoforms. Assuming that the different subfamilies have indeed different functions, could one isoform nevertheless compensate the loss of another? In *Chlamydomonas reinhardtii*, the loss of the conventional actin is compensated by enhanced expression of a highly divergent actin called "novel actin-like protein" (NAP), which is only negligibly expressed in stationary wild type cells [54].

### Phylogenetic distribution

The diversity of *P. tetraurelia *actins and ARPs is well represented in the phylogenetic tree (Fig. 2). While the different actin isoforms of *H. sapiens *and *D. melanogaster*, respectively, build defined branches, most *P. tetraurelia *actins are isolated and scattered throughout the tree. They do hardly cluster with actins from other organisms, not even with those from other ciliates and protozoa (see additional file 3). Exceptions are ARP sequences and sequences with uncertain assignments. Some of them cluster in branches with the corresponding ARP sequences from the other organisms, which emphasize their classification as such. However, apparently unambiguous assignments of those sequences are nevertheless critical due to the above mentioned problems. Also, in an expanded tree using 71 sequences from 26 organisms, the clustering of possible ARPs with ARPs from other organisms is reduced (see additional file 4). The findings agree with the diversity within ciliate actins recently reported from other species by Kim *et al*. [55]. Similarly, ARPs from apicomplexans do not group with any of the known ARP clades [47], although they are considered closely related to ciliates.

### Actin consensus pattern

Most *P. tetraurelia *actins do not contain any of the three actin signatures (Fig. 3), and only five isoforms (actin1-1 to actin1-5) do possess all three. Neither ARPs nor ALP possess the actin consensus pattern which usually serves to detect them. One should keep in mind that these patterns detect most, but not all, actins, ARPs and ALPs in the database. There are a number of known false negative hits when these patterns are used in PROSITE search [31]. The lack of any actin consensus pattern in most of the *P. tetraurelia *isoforms again illustrates the highly divergent character of actins and ARPs in this organism.

### Amino acids influencing polymerization

Several amino acids of the DNase I binding loop, the hydrophobic plug and the C-terminus region are involved in the intermolecular interaction across the actin filament [32,33]. In *P. tetraurelia *actin subfamilies, many of them are lacking (Table 3). This is especially true of the residues in the DNase I binding loop and the hydrophobic plug, which in *Paramecium *are generally not well conserved. Among Spirotricha (ciliates), these regions are segments of increased nonconservative sequence variations [56]. The alteration of the residues H40 and Q41 in the DNase I binding loop may affect filament formation [57]. Both residues are best conserved in subfamily 1, together with the corresponding partners. Concomitantly, immuno-localization with antibodies raised against subfamily 1 showed occurrence of several polymeric structures [12]. Moreover, as the antibodies were designed at a time where not all isoforms had yet been found, it might well be that they also recognize members of subfamily 2 and subfamily 3 (~ 60% and ~ 50% identity in the regions selected for antibody production).

Residues mediating binding between adjacent monomers are also largely altered in several apicomplexans, thus providing an explanation of the absence of stable, long filaments in these parasites [57,58], apart from sequestration of monomers and capping filaments by ABPs [59]. The absence of residues involved in intermonomer contact does not necessarily mean that these isoforms are not able to polymerize. One should keep in mind that the analysis was based only on certain primary structures. It is still possible that, due to the different length of the isoforms, relevant amino acids may match in the respective overall structures with the adjacent monomer. Indeed localization studies with ABs against actin4-1 showed polymerized actin in *Paramecium *cells [53]. Moreover, recall that for several *P. tetraurelia *sequences an explicit classification as actin or ARP is not possible. ARP1 would be the only ARP able to form a homopolymer filament in vivo in other eukaryotes [60].

### ATP binding

Binding and hydrolysis of ATP is important for the function of actin and several ARPs [19,36]. In most actin subfamilies, the nucleotide binding capacity is well conserved (Fig. 4), probably allowing binding and subsequent hydrolyzing of ATP, thus allowing for actin turnover in filaments. The identities of binding residues in the different ARP subfamilies in *P. tetraurelia *are in accordance with the conservation pattern of the respective ARP subfamilies shown in a comparative analysis (Fig. 4; [17]). The lowest residue identity was observed in actin subfamily 7, which also matches as ARP4. Indeed, a recent report suggests that, in yeast, ATP binds weakly to ARP4 [61]. It remains open whether actins/ARPs with a restricted number of conserved residues are able to bind ATP, though with lower affinity, or whether other residues are involved. Interestingly, ATP binding may not be important for nuclear ARPs, as several studies have shown that mutation in nucleotide contact residues of several ARPs did not impair their function [62,63].

### Binding residues for myosin II

Regarding the variety and diversity of *P. tetraurelia *actin isoforms, the question arises whether differences in actin isoform structure determine which ABPs can bind. According to automatic annotations performed with the *Paramecium *database, myosin II is present in the *Paramecium *genome [27]. The myosin II binding site is quite differentially conserved across *P. tetraurelia *subfamilies (Fig. 5). Most subfamilies show conservation between 40 to 60%. The possibility of those isoforms to bind myosin II with either lower affinity or via other residues needs to be clarified. The lowest conservation could be observed in orthologs designated as actin/ARP and ARPs, respectively, which are not supposed to bind myosin II. Several members of subfamily 1 possess all of the necessary residues. Immuno-localization studies with antibodies raised against actin subfamily 1 showed cortical actin in *P. tetraurelia *[12], both on the light and electron microscopic level. Similar results were obtained using fluorescent phalloidin and heavy meromyosin [64]. The interaction of cortical actin with myosin is essential for cyclosis, an actomyosin-based process, also in *Paramecium*.

### Drug binding residues

Surprisingly, for cytochalasins, some of the most common drugs used to influence the dynamics of actin, we could not find any data on putative binding residues. However, for the filament stabilizing drug phalloidin and for the monomer sequestering drug latrunculin A, several studies revealed specific amino acids involved in their binding [39-41]. Jasplakinolide, another potent inducer of actin polymerization, binds actin filaments competitively with phalloidin, presumably due to common binding sites [65]. In *Paramecium*, the binding residues for phalloidin [39,40] are only well conserved in several members of the actin1subfamily (Table 4, Fig. 3). Concomitantly, microinjection of fluorescent phalloidin in *P. tetraurelia *resulted in a distinct labeling pattern [60], which was consistent with localization studies with antibodies against subfamily 1 [12]. However, no staining of the cleavage furrow could ever be achieved, although actin is unequivocally involved in cell division. Indeed, immuno-localization studies with an isoform-specific antibody against actin4, a subfamily lacking the phalloidin binding site, resulted in the labeling of the cleavage furrow [53].

The situation is similar for latrunculin A, where no isoform possesses all binding residues (Table 4, Fig. 3), suggesting low drug binding affinities. In fact, many studies with *Paramecium *had to apply unusually high drug concentrations. Therefore, transferring tools commonly used in other systems to *P. tetraurelia*, may be problematic because the drug cannot bind to the responsible isoform.

## Conclusion

Analysis of all the different features of actin and ARPs in *P. tetraurelia*, on nucleotide and amino acid levels, revealed exceptional differences in comparison to actins and ARPs from other organisms. Also within this organism, members of several subfamilies are considerably different. The basis of this differentiation is likely gene duplication, including a very recent one [29]. This may also explain the occurrence of some silent mutations (with identical derived amino acid sequences) as well as the occurrence, on average, of two very similar members per subfamily. The wide diversification may be reflected by different functions and localization of the actin and ARP subfamily members within a *Paramecium *cell.

## Methods

### Stocks and cultures

The wild-type strains of *P. tetraurelia *used were stock strains 7S and d4-2, derived from stock strain 51S [66]. Cells were cultivated in a decoction of dried lettuce monoxenically inoculated with *Enterobacter aerogenes*, and supplemented with 0.4 μg· ml^-1 ^β-sitosterol [67].

### PCR of genomic DNA and cDNA

Total wild-type DNA from strain *7S *for PCR was prepared from log-phase cultures as published by Godiska *et al*. [68]. The ORFs of individual actin genes were amplified by reverse transcriptase (RT)-PCR using total RNA prepared according to Haynes *et al*. [69]. RT-PCR was performed in a programmable thermocycler T3 (Biometra, Göttingen, Germany) using 3'-oligo dTT primer and the SuperScript™ III reverse transcriptase (Invitrogen, Karlsruhe, Germany) for first-strand cDNA synthesis. 3'-oligo dTT primer containing the artificial restriction sites EcoRI/NotI was: 5'-AACTGGAAGAATTCGCGGCCGCGGAATTTTTTTTTTTTTT-3'. The subsequent PCR reaction (50 μl) was performed with the Advantage 2 cDNA polymerase mix (Clontech, Palo Alto, California) using actin specific oligonucleotides (see additional file 2: oligonucleotides used to study gene expression) with or without the artificial restriction sites XhoI, HindIII or StuI added at their 5'-ends. In general, amplifications were performed with one cycle of denaturation (95°C, 1 min), 40–42 cycles of denaturation (95°C, 30 s), annealing (54–58°C, 45 s) and extension (68°C, 3 min), followed by a final extension step at 68°C for 5 min. PCR products were subcloned into the plasmid pCR2.1 by using the TOPO-TA Cloning Kit (Invitrogen) according to the manufacturer's instructions. After transformation into *E. coli *(DH5 cells or TOP10F' cells), positive clones were sequenced as described below.

### Sequencing

Sequencing was done by MWG Biotech (Ebersberg, Germany) custom sequencing service. DNA sequences were aligned by the CLUSTAL W, integrated in the DNASTAR Lasergene software package (Madison, WI).

### Sequencing of non-radioactive and radioactive probes

Oligonucleotide 1 and 2 were also used to generate non-radioactive and radioactive probes by utilizing the PCR DIG Probe Synthesis Kit from Roche Diagnostics (Mannheim, Germany) or by α-^32^P]dNTP incorporation using a Random Primers Labeling System (Gibco-BRL, Cergy-Pontoise, France), according to the supplier's protocol.

### Hybridization cloning

Hybridization cloning from an indexed library of *Paramecium *macronuclear DNA [28] was carried out as described previously [70]. For the first published actin gene of *P. tetraurelia *[23], 27 positive spots were detected on type 1 filters, seven of which were also analyzed in detail on type 2 filters and sequenced (47F1, 55B19, 87M3, 96J10, 107D11, 139C10, and 149A2 [internal designations; 28]). For another actin gene, named actin2, 10 positive spots were detected on type 1 filters, which were analyzed in detail on type 2 filters (M07D05u, 15M16, 27N16, 27P11, 27P12, 47j22, 74C9, 87H1, 87J8, 93E10, and 134L2).

### Annotation and characterization of actin genes

In order to identify further actin genes in *Paramecium*, the developing *Paramecium *database [71] was screened by using the nucleotide and amino acid sequence of already identified and annotated *Paramecium *actin genes. Positive hits were further analyzed by performing BLAST searches with GenBank at the NCBI database [72]. Additional classification was performed using ARPAnno, an actin-related protein annotation server [18]. Conserved motif searches were performed with either PROSITE [31], or with BLAST-RPS using pfam entries of the corresponding CDD database [73,74]. Phylogenetic and molecular evolutionary analyses were performed with either Clustal W and PhyloDraw (version 0.8; Department of Computer Science, Putan National University, Korea [30], or the Mega version 3 program [75].

## Authors' contributions

RK performed gene fishing and annotation. IMS carried out the molecular studies and drafted the manuscript. EW performed hybridization cloning and cDNA-analysis of act1-1, act1-2, act1-3; JM of act2-1 and act3-1. cDNA-analysis of act1-6, act6-1 and act8-1 was performed by CR. HP and RK conceived this study, and participated in its design and coordination and helped to draft the manuscript. All authors read and approved the final manuscript.

## Supplementary Material

Additional file 1 Alignment report for P. tetraurelia actins, ARPs and ALP. This alignment of all *P. tetraurelia *actins, ARPs and ALPs is the basis of their classification as shown in Table 1, and was used for subsequent sequence analysis (e.g., the presence of specific binding residues). Click here for file

Additional file 2 Oligonucleotides used to study gene expression (cDNA). This table includes all *P. tetraurelia *actin-specific oligonucleotides used for PCR reactions. Click here for file

Additional file 3 Phylogenetic tree composed of sequences from different Protozoa. This phylogenetic tree encompasses sequences for actins, ARPS and ALPS from 13 Protozoa. Click here for file

Additional file 4 Phylogenetic tree composed of sequences from 26 organisms. This phylogenetic tree encompasses 71 sequences for actins, ARPS and ALPS from 26 organisms across all kingdoms. Click here for file

## Figures and Tables

**Table 1:** Molecular characteristics of actins (act), actin-related proteins (ARP), and actin-like protein (ALP) in *P. tetraurelia*

			DNA				Protein			
Gene/subfamily	Accession number	Scaffold number^d^	Length[bp]	*ORF[bp]*	Introns[number]	Identity^a^[%]	Length [aa]	Size [kDa]	Identity^a^[%]	Identity^a,*b*^[%]

*actin1*										
*act1-1*^c^	AJ537442	13	1183	1128	2	100	375	41.7	100	100
*act1-2*^c^	AJ537443	20	1182	1128	2	98.0	375	41.7	100	100
*act1-3*^c^	AJ537444	32	1184	1128	2	94.8	375	41.7	100	100
*act1-4*^c^	AJ537445	111	1179	1128	2	78.6	375	41.7	89.9	89.9
*act1-5*	CR855974	85	1180	1128	2	80.5	375	41.6	91.5	91.5
*act1-6*^c^	CR548612	1	1128	1128	0	65.8	375	41.2	61.7	61.7
*act1-7*^c^	CR855973	8	1128	1128	0	71.5	375	41.7	76.9	76.9
*act1-8*	CR855972	105	1168	1113	2	79.3	370	42.1	70.2	70.2
*act1-9*^c^	CR855988	41	1158	1131	1	75.2	376	43.3	63.3	63.3
*act1-10*^c,*e*^	CR855989	73	1200	1149	1	66.2	103	11.9	10.9	12.2
*actin2*										
*act2-1*^c^	AJ537446	40	1256	1131	5	100	376	42.4	100	59.6
*act 2-2*	AJ537447	102	1257	1131	5	89.9	376	42.4	99.5	59.6
*actin3*										
*act 3-1*^c^	AJ537448	8	1113	1113	0	100	370	42.4	100	44.1
*act 3-2*^c^	CR548612	1	1113	1113	0	82.7	370	42.1	82.7	44.7
*actin4*										
*act 4-1*^c^	CR855971	147	1066	1038	1	100	345	38.5	100	28.2
*act 4-2*	CR856039	128	1062	1038	1	90.8	345	38.5	96.8	28.5
*actin*5(*ARP1)*										
*act 5-1*^c^	CR855970	156	1282	1131	6	100	376	42.8	100	39.6
*act 5-2*	CR855969	20	1284	1131	6	93.5	376	42.8	100	39.6
*act 5-3*	CR855968	13	1284	1131	6	80.3	376	42.9	86.7	39.1
*actin6*										
*act 6-1*^c^	CR855967	52	1296	1185	4	100	394	44.1	100	30.9
*act 6-2*	CR855986	60	1294	1185	4	90.5	394	45.0	93.7	31.1
*actin*7(*ARP2/4)*										
*act 7-1*^c^	CR855941	115	1217	1164	2	100	387	44.9	100	23.7
*act 7-2*	CR855965	113	1217	1164	2	89.3	387	44.8	95.4	25.0
*actin8*										
*act 8-1*^c^	CR548612	1	1100	1056	2	100	351	40.6	100	37.0
*actin9(ARP10)*										
*act 9-1*^c^	CR855964	122	1026	999	1	100	332	38.0	100	17.6
*ARP*2										
*ARP 2-1*	CR855976	14	1314	1176	5	100	391	43.8	100	41.2
*ARP 2-2*	CR855992	42	1309	1176	5	90.7	391	43.9	96.2	40.2
*ARP*3										
*ARP 3-1*	CR856038	62	1401	1278	5	100	426	48.9	100	36.2
*ARP 3-2*	CR855990	24	1401	1278	5	92.6	426	48.9	98.6	36.2
*ALP1*(*ARP*5)										
*ALP 1-1*	CR855977	19	2002	1978	1	100	658	76.9	100	25.0

^a ^Sequences were aligned by Clustal W

^b ^Numbers are referred to the amino acid sequence of act1-1

^c ^Genes are analyzed also on the cDNA level

^d ^*Paramecium *genome project, series of cloned pieces of DNA representing overlapping regions of a particular chromosome, that are in the right order

^e ^Putative pseudogene

**Table 2:** Identities between *P. tetraurelia *actin paralogs and actins from other organisms on amino acid level

% Identity^a ^[aa]	*T. thermophila *[AAP79896]	*T. gondii *[AAC13766]	*D. discoideum *[P02577]	*S. cerevisiae *[NP_116614]	*C. elegans *[CAA34718]	*D. melanogaster *[AAF57294]	*R. norvegicus *[ATRTC]	*A. thaliana *[AAM65277]	*H. sapiens *[AAH16045]
actin1-1	78.5	73.9	73.9	71.8	72.3	73.4	72.3	73.4	72.6
actin1-2	78.5	73.9	73.9	71.8	72.3	73.4	72.3	73.4	72.6
actin1-3	78.5	73.9	73.9	71.8	72.3	73.4	72.3	73.4	72.6
actin1-4	77.4	70.2	69.9	70.2	69.9	70.2	69.7	69.4	69.9
actin1-5	76.3	70.7	70.5	71.0	69.4	70.2	69.7	69.9	69.9
actin1-6	57.7	54.5	56.1	56.1	56.1	56.1	56.1	56.1	56.4
actin1-7	69.1	66.88	64.4	66.5	66.2	64.4	65.4	64.9	65.7
actin1-8	59.8	58.8	57.1	57.4	56.6	57.1	56.6	56.6	56.6
actin1-9	57.0	53.3	52.3	52.5	51.5	52.5	51.7	51.5	52.0
actin2-1	62.6	61.3	60.5	58.6	60.5	60.5	61.8	60.2	62.1
actin3-1	46.9	44.2	45.0	46.6	45.0	44.7	44.7	44.5	44.7
actin4-1	30.6	29.5	30.3	30.6	30.9	30.6	30.6	30.3	30.6
actin5-1(ARP1)	42.2	40.6	40.3	41.6	40.6	41.1	40.6	40.6	40.6
actin6-1	30.6	29.6	30.1	33.2	30.9	31.1	31.4	30.6	31.4
actin7-1(ARP2/4)	24.2	22.9	22.7	24.7	23.7	23.5	23.7	23.7	23.7
actin8-1	38.9	35.6	37.5	38.1	38.6	37.8	38.3	36.9	38.1
actin9-1(ARP10)	19.5	20.4	20.4	20.4	20.1	19.5	19.8	19.8	19.5

^a ^Sequences were aligned by Clustal W method GenBank accession numbers in brackets

**Table 3:** Amino acids influencing polymerization^a^

	***H40/E167***		***Q41/K113 S265 C374***			
actin1-1	***H***	***E***	***Q***	***K***	N	***C***
actin1-4	***H***	***E***	K	*K*	N	*C*
actin1-5	***H***	***E***	***Q***	***K***	H	***C***
actin1-6	Q	*E*	N	*K*	K	*C*
actin1-7	Q	*E*	K	*K*	E	*C*
actin1-8	Y	*E*	D	*K*	***S***	***C***
actin1-9	K	*E*	***Q***	E	K	***C***
actin2-1	Q	*E*	P	A	I	*C*
actin3-1	-	*E*	-	*K*	N	*C*
actin4-1	-	D	-	*K*	N	*C*
actin5-1(ARP1)	Y	D	K	Q	E	Q
actin6-1	N	Q	D	*K*	P	T
actin7-1(ARP2/4)	-	D	-	*K*	P	M
actin8	-	*E*	-	*K*	P	*C*
actin9(ARP10)	-	D	-	E	Y	N
ARP2	A	D	D	*K*	W	V
ARP3	Q	D	K	P	E	A
ALP1(ARP5)	-	D	-	D	*S*	P

^a ^Analysis was performed using Clustal W.

Pairs H40/E167, Q41/K113, Q41/S265, Q41/C374, S265/C374. ***Bold and italic***, both interacting residues are present; *italic*, only one residue is present; dash, no alignment possible

**Table 4:** Drug binding residues

	phalloidin			latrunculin A^b^							
	***G158***	***R177***	***D179***	***D157***	***R183/D184***		***R210/D211***		***K21/E214/K215***		

actin1-1	***G***	***R***	***D***	S	*R*	A	***R***	***D***	***K***	***E***	***K***
actin1-4	***G***	***R***	***D***	S	*R*	A	***R***	***D***	***K***	***E***	***K***
actin1-5	***G***	***R***	***D***	S	*R*	A	***R***	***D***	***K***	***E***	***K***
actin1-6	*G*	K	N	S	*R*	L	K	N	*K*	*E*	R
actin1-7	***G***	***R***	***D***	S	*R*	A	K	*D*	***K***	***E***	***K***
actin1-8	*G*	*R*	Y	S	S	T	***R***	***D***	***K***	***E***	***K***
actin1-9	*G*	*R*	C	C	N	A	D	I	***K***	***E***	***K***
actin2-1	*G*	K	H	S	***R***	***D***	N	A	D	T	*K*
actin3-1	***G***	***R***	***D***	S	Y	A	S	L	Y	V	A
actin4-1	*G*	Q	P	I	E	I	P	*D*	*K*	F	I
actin5-1(ARP1)	***G***	***R***	***D***	V	***R***	***D***	I	I	*K*	Y	*K*
actin6-1	*G*	F	S	L	A	E	D	K	S	*E*	E
actin7-1(ARP2/4)	***G***	***R***	***D***	C	E	Y	S	L	M	Y	G
actin8	*G*	F	N	S	K	S	L	Q	Q	Q	Q
actin9(ARP10)	*G*	G	Y	L	*R*	E	C	Y	D	T	Y
ARP2-1	*G*	*R*	N	A	*R*	H	*R*	E	*K*	*E*	A
ARP3-1	*G*	H	P	S	*R*	D	*R*	E	***K***	***E***	***K***
ALP1-1(ARP5)	*G*	R	N	L	L	N	L	Q	Q	E	R

^a ^Analysis was performed using Clustal W.

^b ^each combination represents an allele in yeast mutagenesis studies.

***Bold and italic***, all interacting residues are present; *italic*, not all residue are present.
