# Supplementary material for: The actin multigene family of Paramecium tetraurelia
Source: BMC Genomics. 2007 Mar 28;8:82. doi: 10.1186/1471-2164-8-82 (PMC1852557; doi:10.1186/1471-2164-8-82)
Supplement: Additional file 2 — Oligonucleotides used to study gene expression (cDNA). This table includes all P. tetraurelia actin-specific oligonucleotides used for PCR reactions. [file 1471-2164-8-82-S2.doc]

| **Additional file 2: Oligonucleotides used to study gene expression (cDNA)** | | |
| --- | --- | --- |
| DNA | Oligonucleotide | Sequence |
| Synthesis of cDNA | | |
| cDNA | 3'-EcoRI/NotI-dTT | 5'-AACTGGAAGAATTCGCGGCCGCGGAATTTTTTTTTTTT-3' |
| Primers used to clone actins from the actin 1 subfamily | | |
| act1-1 | 5'-Act1-1 | 5'-CCGGTATTGCAGGAGATGATG-3' |
| act1-1 | 3'-Act1-1 | 5'-ACCGCTTTCGTCGTATTCG-3' |
| Pairs of actin specific (5') and unspecific (3') primers | | |
| act1-1 | 5'-XhoI-Act1-1  3'-EcoRI/NotI | 5'-CCGCTCGAGATGTCTGAAGAACACCCAGCAG-3'  5'-AACTGGAAGAATTCGCGGCCGCGG-3' |
| act1-2 | 5'-StuI-Act1-2  3'-EcoRI/NotI | 5'-CCGAGGCCTATGTCTGAAGAACACCCAGCAGTTG-3'  5'-AACTGGAAGAATTCGCGGCCGCGG-3' |
| act1-3 | 5'-StuI-Act1-3  3'-EcoRI/NotI | 5'-CCGAGGCCTATGTCTGAAGAACACCCAGCAGTCG-3'  5'-AACTGGAAGAATTCGCGGCCGCGG-3' |
| act1-4 | 5'-HindIII-Act1-4  3'-EcoRI/NotI | 5'-GGGGTTTTAAGCTTAAAGTATCC-3'  5'-AACTGGAAGAATTCGCGGCCGCGG-3' |
| Pairs of actin specific (5' and 3') primers | | |
| act1-6 | 5'-StuI-Act1-6  3'-XhoI-Act1-6 | 5'-AAAAGGCCTATGTAAGCTTAATATCCAGC-3'  5'-CCGCTCGAGTCAGAAACATTTTCTGTGAAC-3' |
| act1-7 | 5'-StuI-Act1-7  3'-XhoI-Act1-7 | 5'-GAAGGCCTATGTCAGATTAATTACCAGCAGTTATAAT-3'  5'-CCGCTCGAGCAATGAGTAACTCCATCTCCTGAATCG-3' |
| act1-9 | 5'-StuI-Act1-9  3'-XhoI-Act1-9 | 5'-GAAGGCCTATGAATGATGAAAAACCAGCAGTCG-3'  5'-CCGCTCGAGTCAAGTGACTGTCTAACATTTTCTGTG-3' |
| act2-1 | 5’bAStu | 5'-AAGGCCTATGGACGACGTAATCCCAGTTGTG |
|  | 3’bAX | 5'-CCGCTCGAGTCAGAAGCATTTTCTGTGCACATAACC |
| act3-1 | 5’A3  3’A3X | 5'-GTAATTGAAAATGCTTCTTGC  5'-CCGCTCGAGTCAGAAACATTTAATATGTGC |
| act3-2 | 5’Stu-Act3-2  3’Xba-Act3-2 | 5'-GAAGGCCTATGATAGAATCTCATCCTCCTGTTG  5'-GCTCTAGATCAAAAACATTTAATGTGAGCAATC |
| act4-1 | 5’Stu-Act4-1  3’Xho-Act4-1 | 5'-GAAGGCCTATGAATGATGAAAAACCAGCAG  5'-CCGCTCGAGTCAAGTGACTGTCTAAGATTTTC |
| act5-1 | 5’Stu-Act5-1  3’Xho-Act5-1 | 5'-AGGCCTATGGATAATGACATATTTGCTAATAACTCG  5'-ccgctcgagtcacaattattttttgattaaaatg |
| acti6-1 | 5’Spe-Act6-1  3’Xho-Act6-1 | 5'-GCGACTAGTATGGAAAGTGAGTATGACTAAAAAG  5'-CCGCTCGAGTCAAAATGTTCTCTTATGAATAAG |
| act7-1 | 5’Stu-Act7-1  3’Xho-Act7-1 | 5'-GAAGGCCTATGTTCATACCATACAAAAAAAATAAAGGG  5'-CCGCTCGAGTCAATAAAACATTTTTTTTTCTATTAG |
| act8-1 | 5’Stu-Act8-1  3’Xho-Act8-1 | 5'-GAAGGCCTATGAATAATAATGATTCACCTTCTATTA  5'-CCGCTCGAGTCAAAAGCACTTTCTTTATACTA |
| act9-1 | 5’Stu-Act9-1  3’Spe-Act9-1 | 5'-GAAGGCCTATGAGTCTAGACAAATAATCAAGG  5'-GGACTAGTTCACGGTTTAATAGAAATAAAA |
