# Supplementary material for: The actin multigene family of Paramecium tetraurelia
Source: BMC Genomics. 2007 Mar 28;8:82. doi: 10.1186/1471-2164-8-82 (PMC1852557; doi:10.1186/1471-2164-8-82)
Supplement: Additional file 3 — Phylogenetic tree composed of sequences from different Protozoa. This phylogenetic tree encompasses sequences for actins, ARPS and ALPS from 13 Protozoa. [file 1471-2164-8-82-S3.pdf]

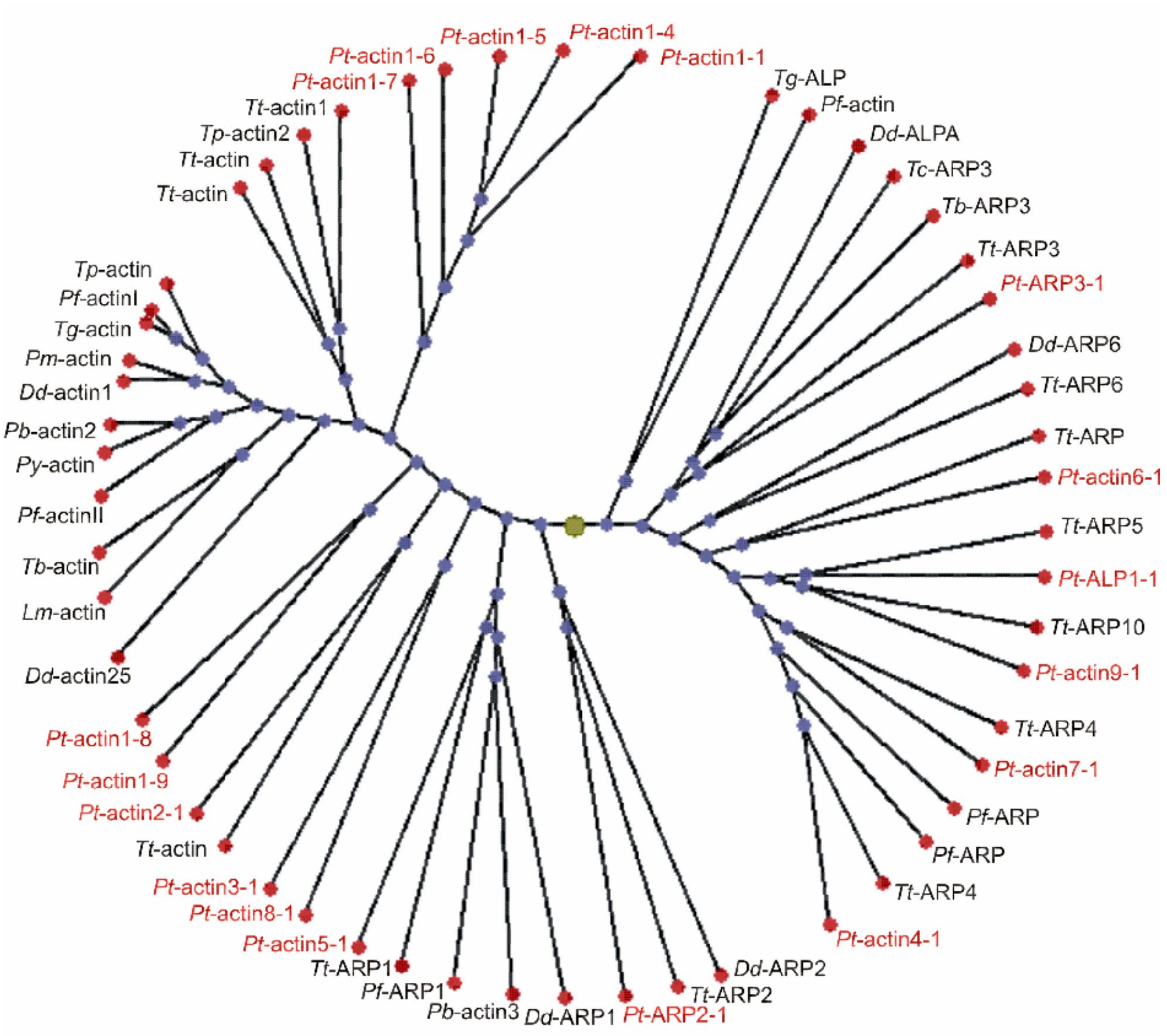

Additional file 3: This phylogenetic tree encompasses *Dictyostelium discoideum* actin1 [GenBank:XP_636169], actin25 [GenBank:XP_636188], ARP1 [GenBank:XP_636500], ARP2 [GenBank:XP_645275], ARP6 [GenBank:XP_637435], ALPA [GenBank:XP_638880]; *Leishmania major* actin a [GenBank:CAC22667]; *Paramecium tetraurelia* act1-1 [GenBank:CAD60960], act1-4 [GenBank:CAD60963], act1-5 [GenBank:CAH69678], act1-6 [GenBank:CAH03399], act1-7 [GenBank:CAH69677], act1-8 [GenBank:CAH69676], act1-9 [GenBank:CAH69752], act2-1 [GenBank:CAD60964], act3-1 [GenBank:CAD60966], act4-1 [GenBank:CAH69675], act5-1(arp1-1) [GenBank:CAH69674], act6-1 [GenBank:CAH69671], act7-1 (arp4-1) GenBank:CAH74221], act8-1 [GenBank:CAH03397], act9-1 (arp10) [GenBank:CAH69669], alp1-1 (arp5) [GenBank:CAH69680], arp2-1 [GenBank:CAH69679], arp3-1

[GenBank:CAH74222]; *Perkinsus marinus* actin [GenBank:AAR11389] ,*Plasmodium berghei* actin2 [GenBank:XP_680164], actin3 [GenBank:CAC48194]; *Plasmodium falciparum* actin [GenBank:NP_703241], actin I [GenBank:AAA29465], actin II [GenBank:AAA29467], ARP1 [GenBank:NP_700976], ARP4 [GenBank:NP_702107], ARP [GenBank:NP_703397]; *Plasmodium yoelii yoelii* actin ii [GenBank:XP_729324]; *Tetrahymena pyriformis* actin2 [GenBank:CAA28824]; *Tetrahymena thermophila* actin2 [Tt9018], ARP [GenBank:AAN73251], ARP1 [Tt17635], ARP2 [GenBank:AAN73249], ARP3 GenBank: [AAN73250], ARP4 [Tt21000], ARP4 [Tt28168], ARP5 [Tt24270], ARP6 [Tt27636], ARP10 [Tt15617]; *Theileria parva* actin [GenBank:EAN33188]; *Toxoplasma gondii* actin [GenBank:AAC13766], ALP [GenBank:AAW23163]; *Trypanosoma brucei* actin [GenBank:AAA30151], ARP3 [EAN76600]; and *Trypanosoma cruci* ARP3 [EAN98434].
