# Supplementary material for: The actin multigene family of Paramecium tetraurelia
Source: BMC Genomics. 2007 Mar 28;8:82. doi: 10.1186/1471-2164-8-82 (PMC1852557; doi:10.1186/1471-2164-8-82)
Supplement: Additional file 4 — Phylogenetic tree composed of sequences from 26 organisms. This phylogenetic tree encompasses 71 sequences for actins, ARPS and ALPS from 26 organisms across all kingdoms. [file 1471-2164-8-82-S4.pdf]

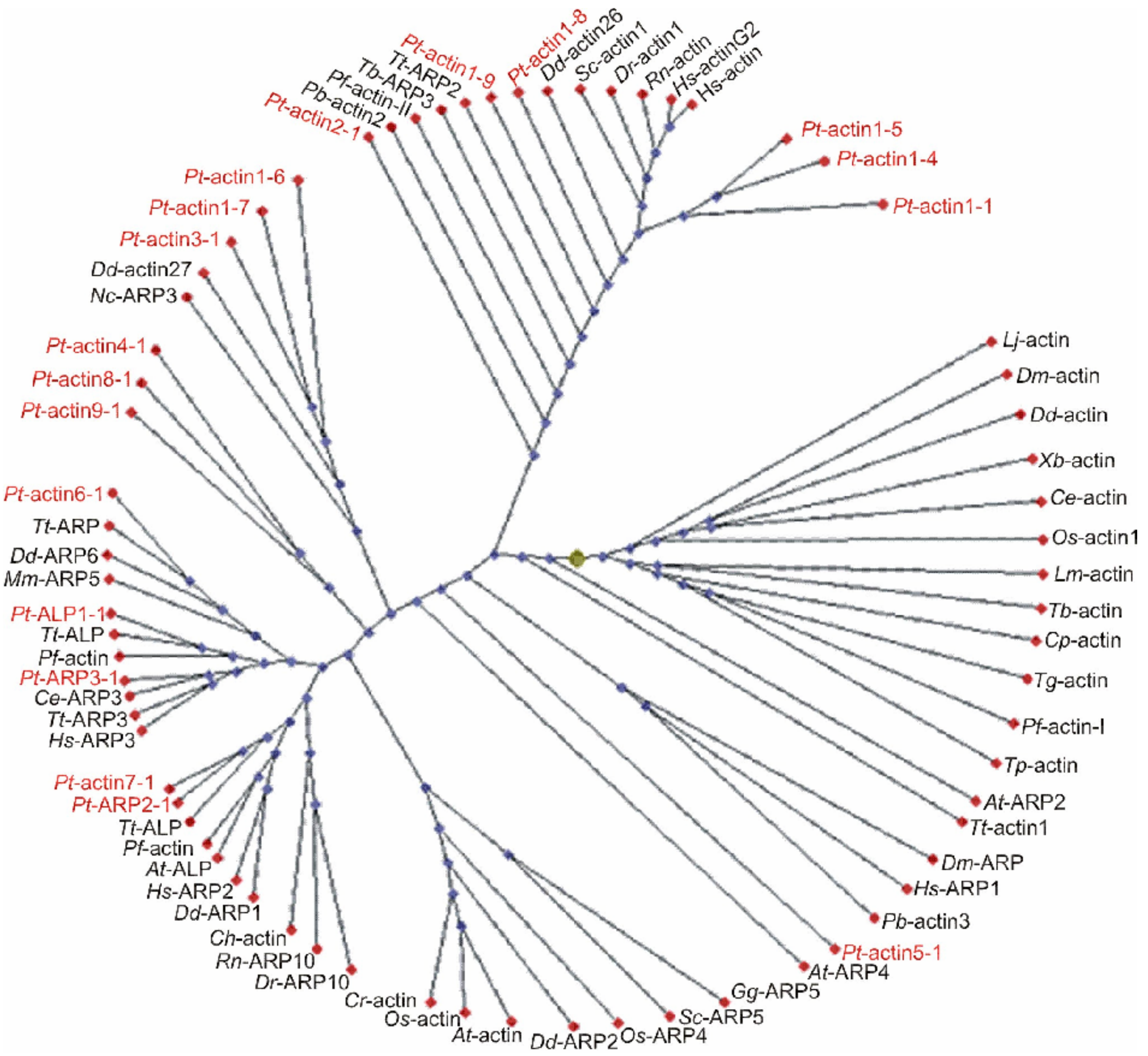

Additional file 4: This phylogenetic tree encompasses *Arabidopsis thaliana* actin [GenBank:AAM65277], ARP2 [GenBank:AAC69601], ARP4 [GenBank AAM53244] and ALP [GenBank:ARP5] [GenBank:BAB03145]; *Caenorhabditis elegans* actin [GenBank:CAA34718] and ARP3 [GenBank:AAF36012]; *Chlamydomonas reinhardtii* actin [GenBank:BAA09449]; *Cryptosporidium hominis* actin [GenBank:XP_667340]; *Cryptosporidium parvum* actin [GenBank:AAA28295]; *Danio rerio* ARP1 [GenBank:NP_998537] and ARP10 [GenBank:AAH45412]; *Dictyostelium discoideum* actin [GenBank:P02577], actin26 [GenBank:XP_646389], actin27 [GenBank:XP_636189], ARP1 [GenBank:XP_636500], ARP2 [GenBank:XP_645275] and ARP6 [GenBank:XP_637435]; *Drosophila melanogaster* ARP [GenBank:CAA55240], actin, [GenBank:BAA20058] and

actin [GenBank:AAF57294]; *Gallus gallus* ARP5 [GenBank:NP_001008446]; *Homo sapiens* actG2[GenBank:CAG38753], ß-actin [GenBank:AAH16045], ARP1 [GenBank:AAH06372], ARP2 [GenBank:NP_005713] and ARP3 [GenBank:NP_005712]; *Laminaria japonica* actin [GenBank ABB80121]; *Leishmania major* actin a [GenBank:CAC22667]; *Mus musculus* ARP5 [GenBank:AAH52039]; *Neurospora crassa* ARP3 [GenBank:AAC78497]; *Oryza sativa* actin1 [GenBank:XP_475316] and ARP4 [GenBank:XP_479987]; *Paramecium tetraurelia* act1-1 [GenBank:CAD60960], act1-4 [GenBank:CAD60963], act1-5 [GenBank:CAH69678], act1-6 [GenBank:CAH03399], act1-7 [GenBank:CAH69677], act1-8 [GenBank:CAH69676], act1-9 [GenBank:CAH69752], act2-1 [GenBank:CAD60964], act3-1 [GenBank:CAD60966], act4-1 [GenBank:CAH69675], act5-1(ARP1-1) [GenBank:CAH69674], act6-1 [GenBank:CAH69671], act7-1 (ARP4-1) GenBank:CAH74221], act8-1 [GenBank:CAH03397], act9-1 (ARP10) [GenBank:CAH69669], ALP1-1 (ARP5) [GenBank:CAH69680], ARP2-1 [GenBank:CAH69679] and ARP3-1 [GenBank:CAH74222]; *Plasmodium berghei* actin2 [GenBank:XP_680164] and actin3 [GenBank:CAC48194]; *Plasmodium falciparum* actin [GenBank:NP_700976], actin I [GenBank:AAA29465], actin II [GenBank:AAA29467] and actin(ARP1) [GenBank:NP_703241]; *Rattus norvegicus* ß-actin [GenBank:ATRTC] and ARP10 [GenBank:AAH87143]; *Saccharomyces cerevisiae* act1p [GenBank:NP_116614] and ARP5 [GenBank:CAA95933]; *Tetrahymena thermophila* actin1 [GenBank:AAP79896], ARP [GenBank:AAN73251], ARP2 [GenBank:AAN73249] ARP3 [GenBank:AAN73250], actin family protein [GenBank:EAS01136] and actin family protein [GenBank:EAR99381]; *Theileria parva* actin [GenBank:EAN33188]; *Toxoplasma gondii* actin [GenBank:AAC13766]; *Trypanosoma brucei* actin [GenBank:AAA30151] and ARP3 [GenBank:EAN76600]; and *Xenopus borealis* actin [GenBank:CAA30390].
